# Endovascular Aortic Repair After Abdominal Aortic Injury in a Patient With an Aberrant Renal Artery

**DOI:** 10.7759/cureus.31450

**Published:** 2022-11-13

**Authors:** Jernej Lučev, Silva Breznik, Biljana Lamanovska, Pia Šumer, Aleš Slanič

**Affiliations:** 1 Department of Radiology, University Medical Centre Maribor, Maribor, SVN

**Keywords:** interventional radiology, dissection, blunt trauma, endovascular aortic repair, abdominal aortic injury

## Abstract

Abdominal aortic injury (AAI) due to blunt trauma is rare and is often complicated by thrombosis within the true and false lumens and sometimes aortic rupture. No standard guidelines for treatment are available. We present the case of a 44-year-old female patient with posttraumatic dissection of the abdominal aorta, which was referred to our institution for endovascular aortic repair (EVAR). The patient was referred to our institution after emergency surgery following blunt abdominal trauma due to a car accident. Initial computed tomography (CT), performed at the referring hospital, showed multiple bone injuries with pneumothorax, liver and spleen lacerations, and rupture of the anterior abdominal wall with mesenteric injury and active intraperitoneal extravasation of contrast media from visceral arteries. Initial CT also showed dissection of the distal part of the abdominal aorta. Due to hemodynamic instability, emergency surgery was performed for intraperitoneal injuries. Control computed tomography angiography (CTA) after surgery confirmed a dissection of the distal part of the abdominal aorta at the level of the bifurcation protruding into the right common iliac artery with partial thrombosis of the right iliac artery and no active extravasation of the contrast media at the level of the aorta. An aberrant left renal artery was also identified.

A hemodynamically stable patient was transferred to our institution for emergency EVAR which was performed without intraprocedural complications. Control CTA after EVAR showed a good result of the procedure with minimal type 2 endoleak and no extravasation. EVAR can also be used to treat AAI without active extravasation to prevent future total rupture of the aortic wall.

## Introduction

Abdominal aortic injury (AAI) is rare but historically associated with significant morbidity and mortality [1]. It occurs only in 0.03%-0.1% of blunt trauma patients [2]. Although its incidence is less common than thoracic aorta injury, which is reported to be 1.5%-2%, it can be life-threatening if not treated appropriately [2-4].

As a result of trauma, AAI is usually not isolated and is often seen in combination with spine injuries, intraperitoneal and retroperitoneal organ injuries, and abdominal wall injuries [5,6]. No standard guidelines for the treatment of AAI are available [7]. Published data suggest that conservative treatment with patient observation in the case of small pseudoaneurysms and intimal flaps appears safe [8]. They also report that survival rates are reasonable and that aortic-related complications are rare [8]. The indications for aortic repair in the setting of concomitant bowel injuries are not well defined [8].

As AAI is extremely rare, no established treatment guidelines exist, and the therapy is mainly dependent on the decision of the multidisciplinary team (MDT) [7].

## Case presentation

**Figure 1:**
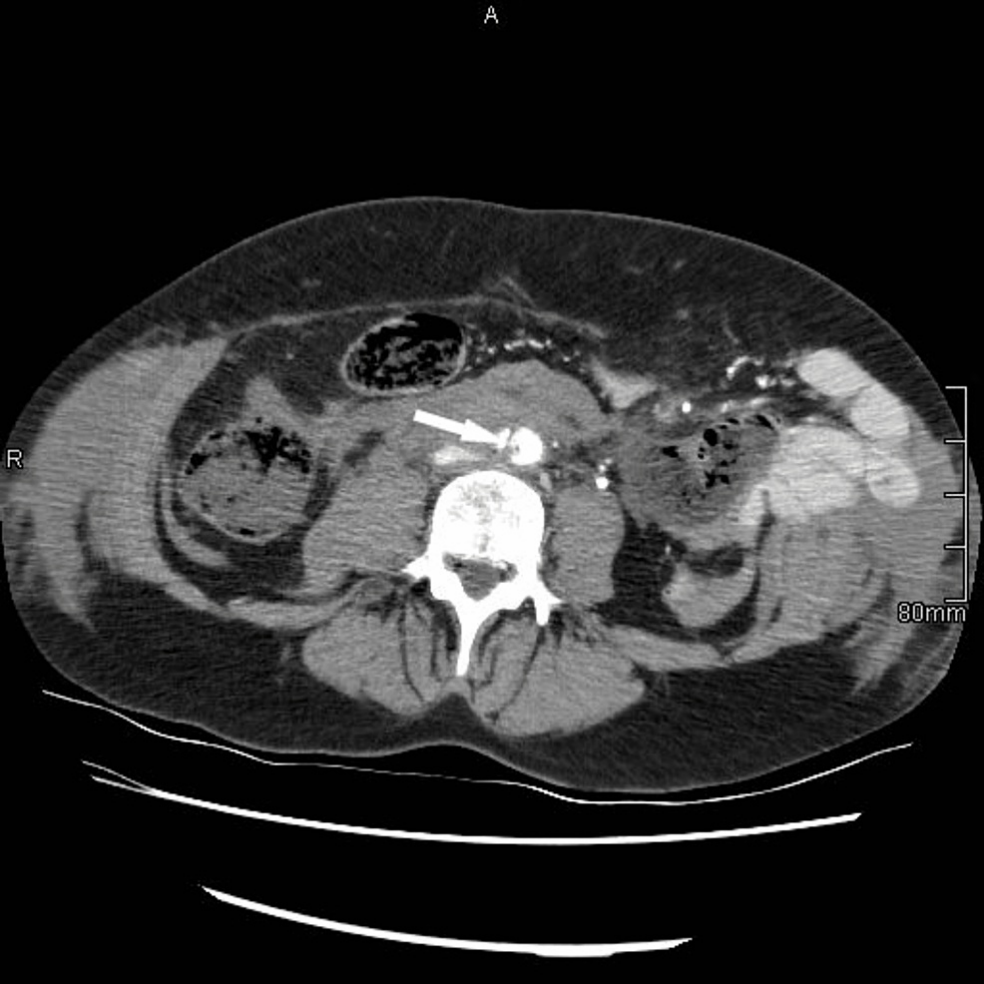
Initial CT, axial image, images show rupture of the anterior abdominal wall with mesenteric injury and dissection (arrow) of the distal part of the abdominal aorta. Periaortic changes are not hematoma but, as confirmed during surgery, displaced bowel loops

A 44-year-old female patient was referred to our hospital for endovascular aortic repair (EVAR) after emergency surgery due to acute intraabdominal bleeding from a mesenteric injury resulting from a car accident. The initial computed tomography (CT) images showed multiple rib fractures with pneumothorax, L2 fracture, liver and spleen lacerations, and rupture of the anterior abdominal wall with mesenteric injury and active intraperitoneal extravasation of contrast media from visceral arteries. CT also showed a dissection of the distal part of the abdominal aorta with no active extravasation (Figure 1).

**Figure 2:**
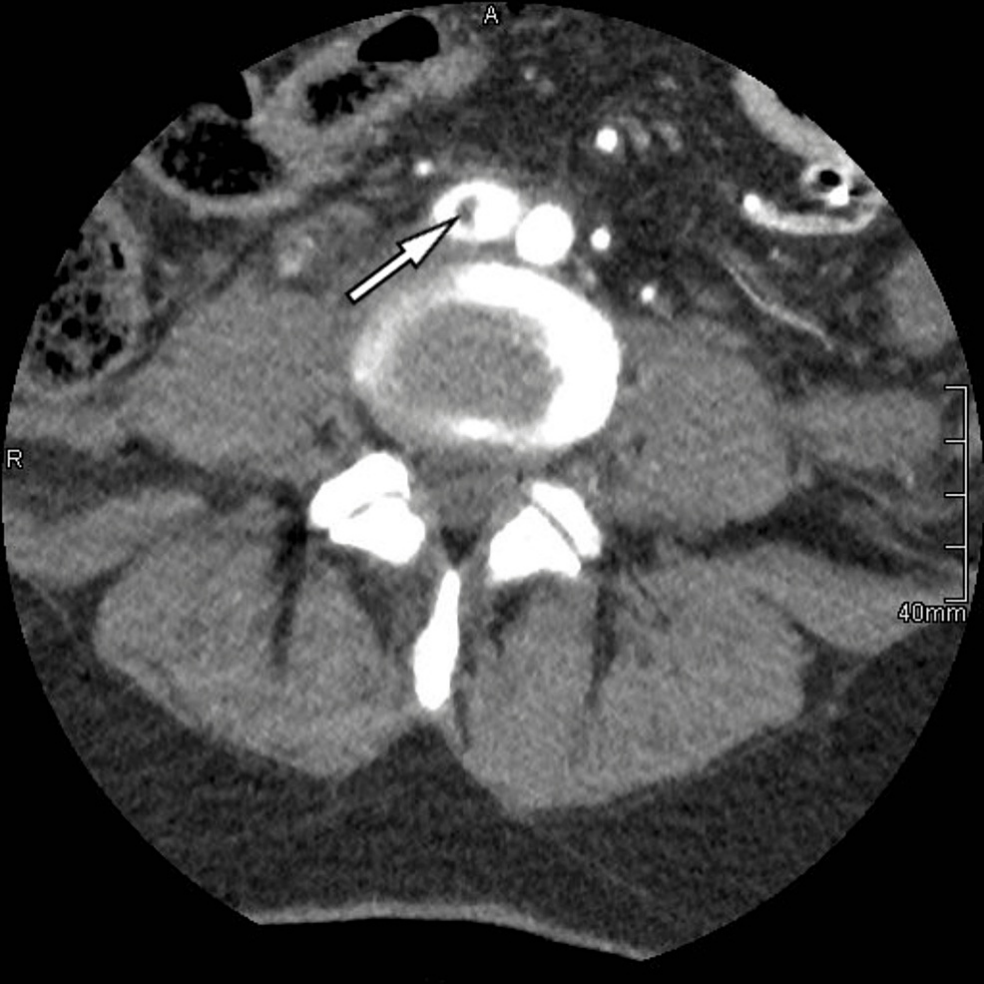
Computed tomography angiography after surgery, axial image, partial thrombosis of the right iliac artery (arrow)

**Figure 3:**
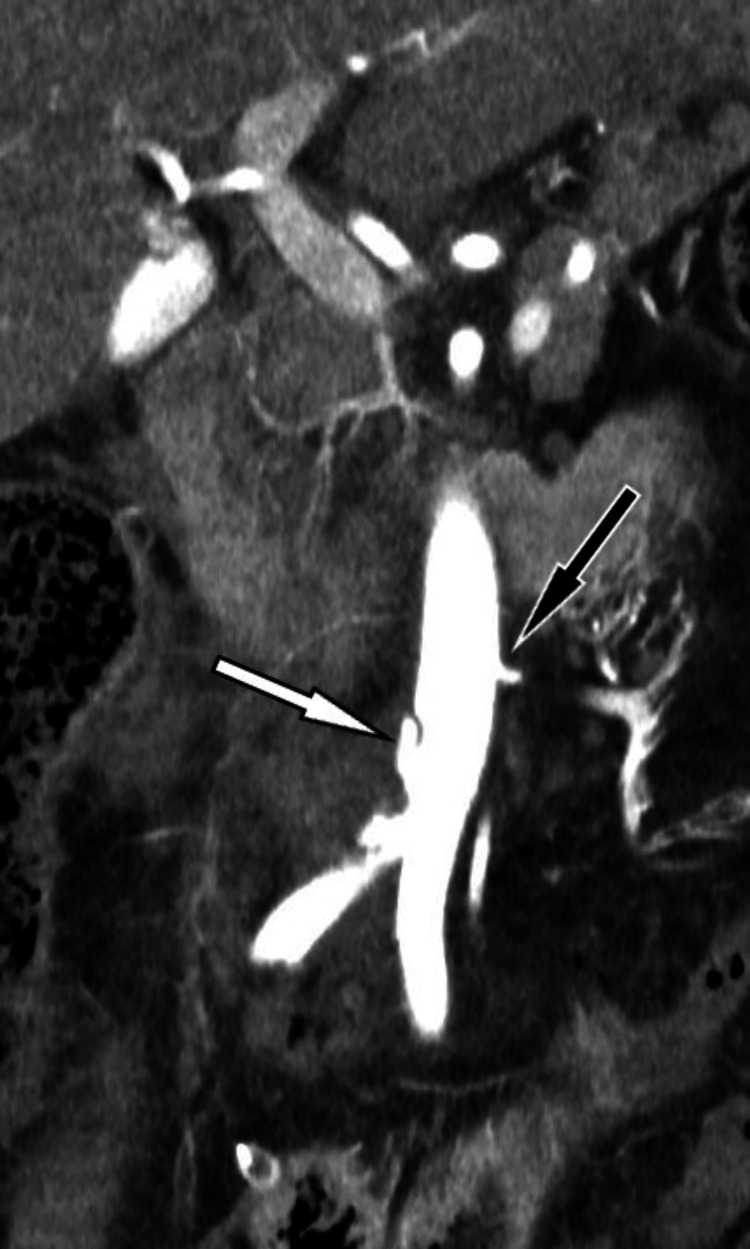
Computed tomography angiography after surgery, coronal image, dissection of the distal part of the abdominal aorta (white arrow) and the left aberrant renal artery (black arrow)

Emergency surgery was performed with small bowel resection, anastomosis, hemostasis, and mesenteric and anterior abdominal wall reconstruction. After the surgery, the patient was hemodynamically stable. Prior to admission to our institution, control computed tomography angiography (CTA) images were acquired according to the instructions of our on-duty radiologist, who also inspected the images in consultation with the on-call interventional radiologist. In addition to multiple bone injuries with postoperative changes in the intraperitoneal space, CTA also confirmed a dissection of the distal part of the abdominal aorta at the level of the bifurcation protruding into the right common iliac artery with partial thrombosis of the right iliac artery and no active extravasation of the contrast media at the level of the aorta (Figure 2). The CTA scans also identified an aberrant left renal artery (Figure 3).

**Figure 4:**
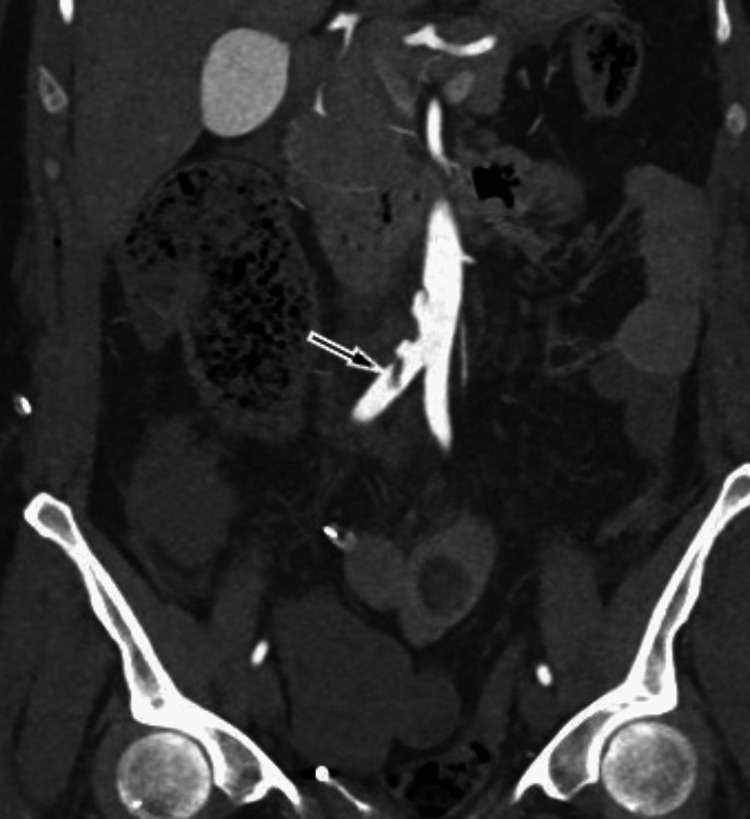
Control computed tomography angiography before EVAR, coronal image, slight progression of the thrombus in the right common iliac artery (arrow) EVAR: endovascular aortic repair.

The patient was transferred to our institution for further treatment. At admission, the patient was ventilated (FiO2 0.5, SpO2 99%) and hemodynamically stable (RR 120/60 mmHg, pulse 75/min), with no critical limb ischemia. Due to the complexity of the case, an MDT was established consisting of two interventional radiologists, an anesthesiologist, and a vascular and abdominal surgent. The MDT decided on another control CTA, which showed slight progression of the thrombus in the right common iliac artery (Figure 4). Treatment options and complications options were discussed in detail and the MDT decided that endovascular treatment should be considered first.

**Figure 5:**
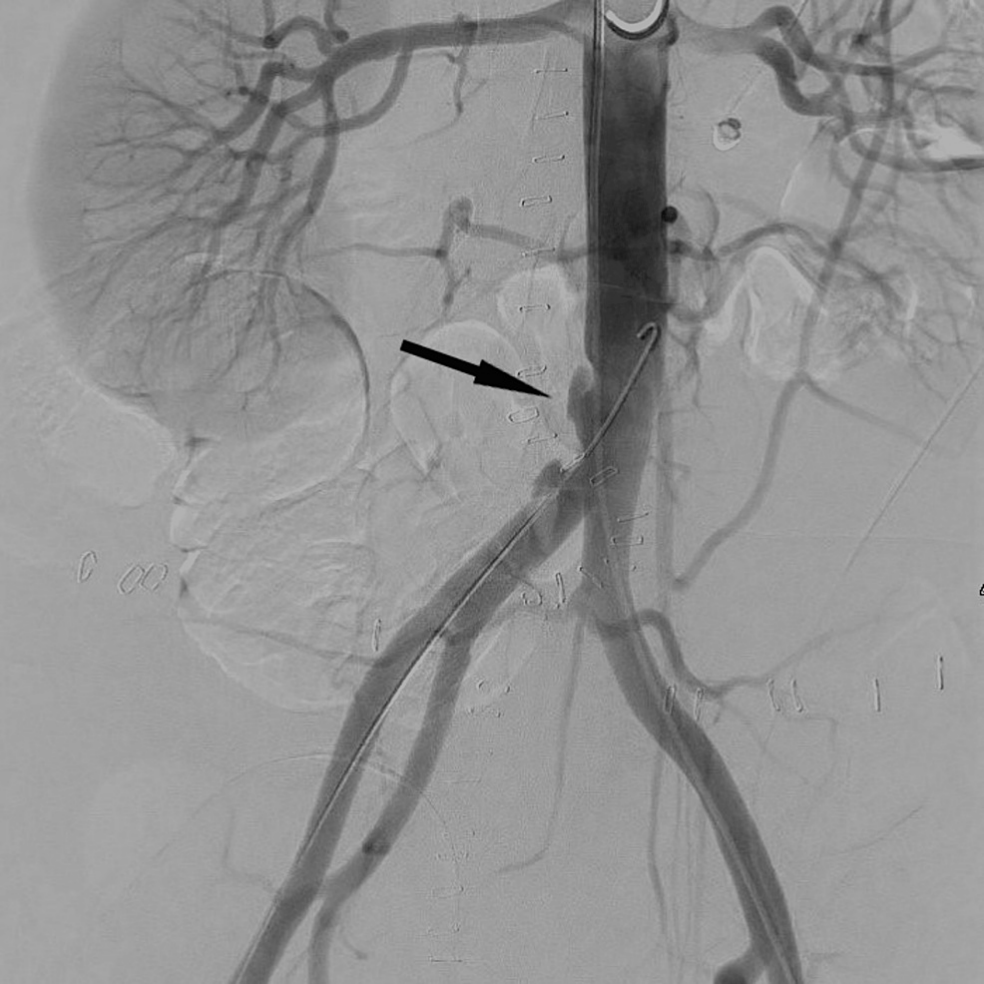
Preprocedural DSA showing dissections of the distal part of the abdominal aorta (arrow) DSA: digital subtraction angiography.

**Figure 6:**
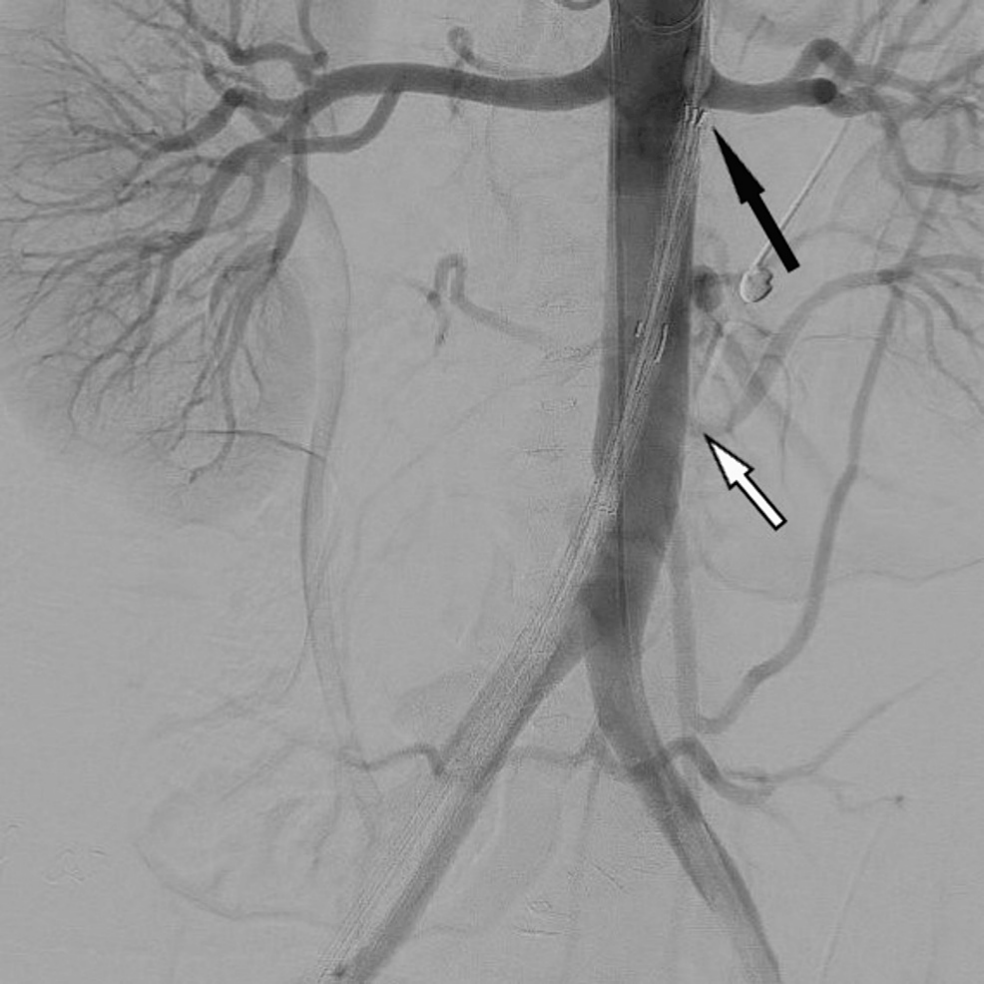
DSA before the deployment of EVAR showing placement below the left renal artery (black arrow) and the position of the left aberrant renal artery (white arrow) DSA: digital subtraction angiography; EVAR: endovascular aortic repair.

**Figure 7:**
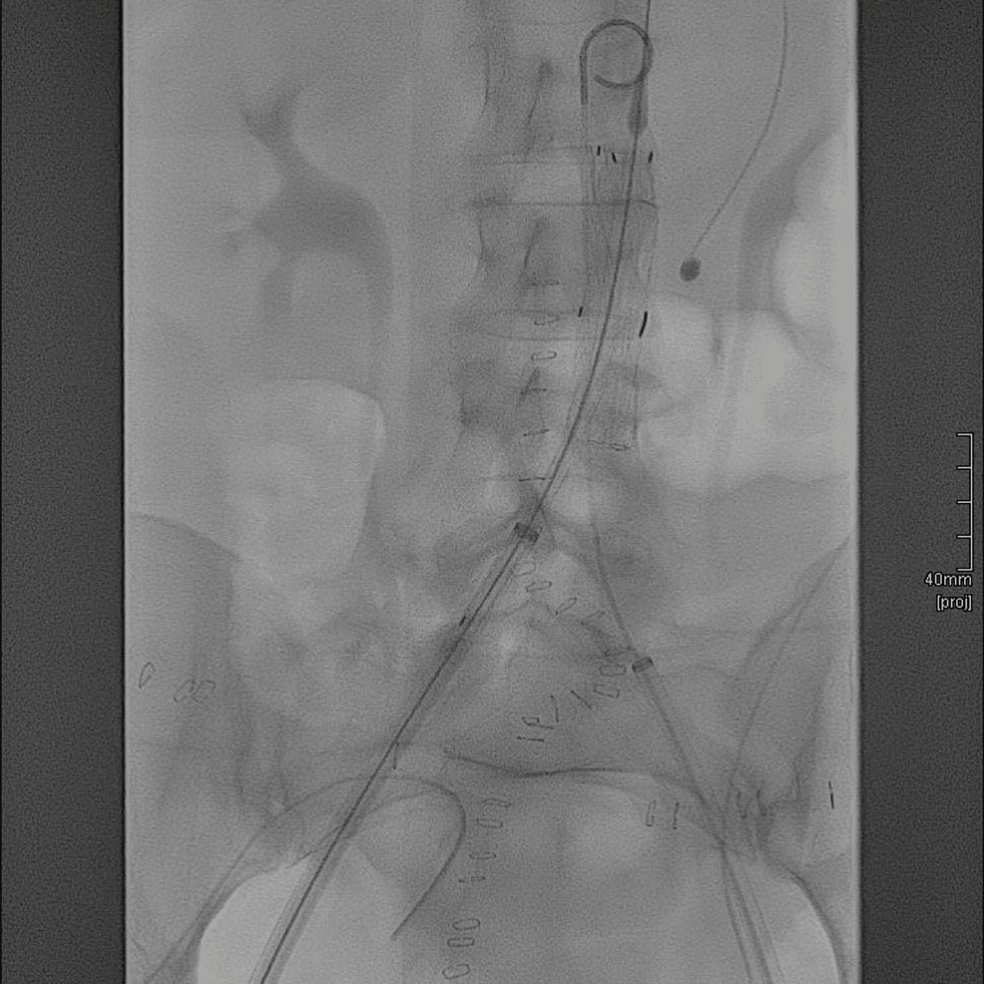
Fluoroscopy during EVAR deployment EVAR: endovascular aortic repair.

**Figure 8:**
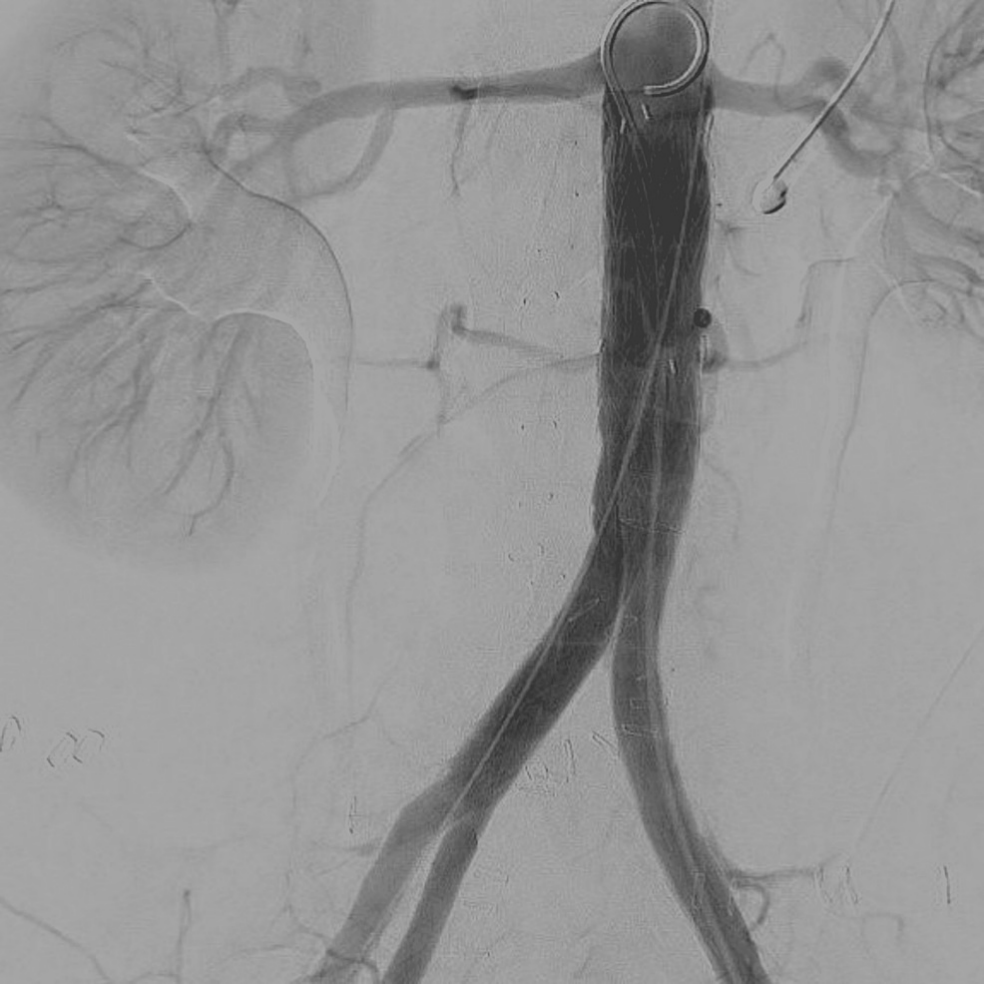
Control DSA after EVAR showing a good result with minimal type 2 endoleak DSA: digital subtraction angiography; EVAR: endovascular aortic repair.

**Figure 9:**
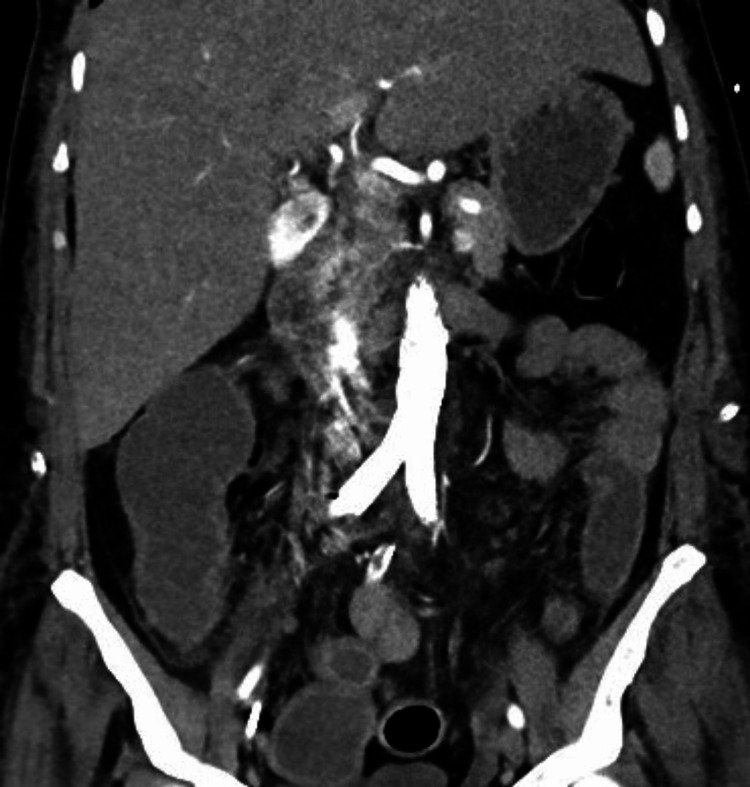
Control computed tomography angiography, coronal image, after the procedure, coronal view, showing a good procedure result with no endoleak and extravasation

The procedure was performed under general anesthesia. Retrograde puncture of the common femoral artery was performed on both sides under ultrasound (US) guidance. Vascular access was secured with a 0.035-inch extra-stiff guide wire, a 16 French sheath on the right side, and a 12 French sheath on the left side. EVAR [Treo (Terumo, Tokyo, Japan)] was deployed below the left main renal artery cowering the left aberrant renal artery (Figure 5-7). The procedure was performed without intraprocedural complications. Control digital subtraction angiography (DSA) after EVAR showed a good result with a small type 2 endoleak (Figure 8). Control CTA after two days showed a good result of the procedure with no endoleak and no extravasation (Figure 9). Malperfusion of the inferior pole of the left kidney was observed on the CT images; however, kidney function parameters were normal (creatinine 50 µmol/L).

## Discussion

Due to blunt trauma, AAI is rare and appears in less than 0.1% [2]. The most common cause is motor vehicle accidents, with an incidence of 57.5% and mortality rates of 28%-73% at discharge [3,9,10]. The most significant predictor of mortality in patients with AAI is hemopneumothorax, followed by inferior vena cava injury [7].

Early diagnosis and selecting the best treatment option for the patient are essential to improve the survival rate and prognosis [7]. If AAI is not diagnosed in time or the treatment is delayed, it can lead to death due to hemorrhage or ischemia of organs [7]. Flow-limiting conditions of the visceral vessels and lower limbs can be overlooked without contrast-enhanced CTA. These misdiagnosed conditions can cause acute mesenteric ischemia or acute limb ischemia in the acute phase and intermittent claudication or chronic mesenteric ischemia in the chronic phase if not treated [8,11].

There are three treatment options for AAI following blunt trauma. The first is conservative treatment with blood pressure reduction, followed by surgical and endovascular treatment with EVAR [8,12].

Patients with minimal aortic injury (MAI) limited to the intima are at low risk of complications and may be considered for observation [1]. Conservative treatment is also successful in uncomplicated cases without external aortic contour abnormality on CT [2].

Patients with severe aortic injury (SAI) manifest clinically or radiographically at submission. Those not associated with bleeding, malperfusion, or thromboembolism may be observed with interval imaging. For all observed patients, long-term surveillance is required to document complete resolution or stability because even MAI can progress to a more complex lesion [1]. Patients with SAI were associated with a significantly higher mortality rate than MAI (50% vs. 0%; p=0.03), as reported by Harris et al. [1]. Other studies suggest that in the case of the dissection of the abdominal aorta caused by blunt trauma, the mortality rate with conservative treatment is 75%. In contrast, it ranges from 18% to 37% with surgical treatment [5].

High mortality rates are reported in free aortic ruptures, which need invasive treatment [2]. Open repair of AAI is still the standard of care, although it is associated with high mortality [12]. Endovascular treatment has enormously evolved in recent years, and studies suggest improved survival rates in appropriately selected patients [12]. It is a safe and efficient method for treating traumatic infrarenal aortic dissection without ischemic paraplegia or other injuries requiring emergency surgery [5]. Dayama et al. reported mortality rates for endovascular treatment vs. surgery of 21% vs. 67%, respectively [12].

In our case, the patient had a dissection at the aortic bifurcation, progressing into the right common iliac artery, which could be classified as MAI and observed. However, she also had a floating thrombus in the proximal part of the right common iliac artery. The thrombus was not flow-limiting but represented a severe threat to future thromboembolic events. Concerning the young age of the patient in combination with long-term surveillance, which would be required to document the complete resolution or stability of the injury, the MDT decided that invasive treatment would be a better option for her. Taking into account the polytraumatic state of the patient and "hostile abdomen" after the initial surgery, we decided that endovascular treatment should be considered first, despite the polar artery of the left kidney. As Lareyre et al. demonstrated, EVAR does not significantly impair early renal postoperative function, suggesting the safety of the procedure [13].

## Conclusions

We reported a case of AAI (dissection) due to blunt abdominal trauma complicated with partial thrombosis of the right common iliac artery. Due to mesenteric bleeding, emergency surgery at the referring hospital was performed before the referral. At referral, an MDT decided that endovascular treatment of the aortic injury would be the best treatment option for the patient, despite the polar artery of the left kidney. EVAR allowed minimally invasive simultaneous treatment of aortic dissection together with thrombus stabilization and minimal future requirements for surveillance.
